# Physiological and proteomic analysis on long-term drought resistance of cassava (*Manihot esculenta* Crantz)

**DOI:** 10.1038/s41598-018-35711-x

**Published:** 2018-12-19

**Authors:** Zhongying Shan, Xinglu Luo, Maogui Wei, Tangwei Huang, Aziz Khan, Yanmei Zhu

**Affiliations:** 1 College of Agronomy, Guangxi University, Nanning, 530004 China; 2 State Key Laboratory for Conservation and Utilization of Subtropical Agro-bioresources, Nanning, 530004 China

## Abstract

Drought stress is one of the potent abiotic stress limiting cassava (*Manihot esculenta*) yield globally, but studies addressing both physiological and proteomic responses that how cassava crops can adjust their growth and metabolism under drought conditions are lacking. Combining leaf physiological and proteomic characteristics strongly allied with drought tolerance should results in enhanced drought tolerance in cassava crop. Therefore, the aims of this study were to explore the plant physiological and proteomic mechanisms involved in drought adaptation in cassava. Xinxuan 048 (XX048) was exposed to well-watered control (CK, relative soil water content (RSWC) as 80 ± 5%), mild drought stress (LD, RSWC as 65 ± 5%), moderate drought stress (MD, RSWC as 50 ± 5%) and severe drought stress (SD, RSWC as 35 ± 5%) from 30 days after planting. Under drought stress conditions, cassava plant showed a substantial decline in plant height, stem diameter, leaf number, leaf water content, the ratio of free water content to bound water content of leaf (FW/BW), net photosynthetic rate (*Pn*), intercellular CO_2_ concentration (*Ci*), stomatal conductance (*Gs*) and transpiration rate (*Tr*) compared with well watered plants. However, compared with control, leaf water content, SPAD value, cell membrane permeability, malondialdehyde (MDA), soluble sugar, protein proline content SOD and CAT activity were at peak under drought stress. The proteomic analysis revealed that among 3 339 identified proteins, drought stress increased and decreased abundance of 262 and 296 proteins, respectively, compared with control condition. These proteins were involved in carbohydrate energy metabolism, protein homeostasis, transcription, cell structure, cell membrane transport, signal transduction, stress and defense responses. These data not only provides a comprehensive dataset on overall proteomic changes in cassava leaves under drought stress, but also highlights the mechanisms by which euphorbiaceae plants can adapt to drought conditions.

## Introduction

Drought was considered as one of the most important abiotic stresses that intimidate the plants’ survival including crops^1^. Water deficient stress could be increased with the global warming due to augmentation in evapotranspiration amounts, while on another side will also intensify the drought stress occurrence as well as their intensity by 2100 with an upsurge from 1 to 30% in acute drought land area^2^, which interrupt the positive effects from the increased CO_2_ concentration. As water assets and arable land become restraining, drought tolerant development in crops and utilization of marginal lands for rising crops will become progressively imperative.

As the sixth most important crop, cassava (*Manihot esculenta* Crantz) is a particularly important staple crop and cash crop of the resource-limited farmers in tropical and subtropical areas, owing to its ability to produce in marginal lands where drought and soil fertility are the main constraints of crop productivity. Although cassava shows strong tolerance to marginal environments, its production is still constrained by drought. The actual yield of cassava in farmers’ field is around 8-fold lower than the highest potential yield when the traditional varieties were used and cultivated on marginal lands without inputs^3^. Some cassava genotypes were found that are well adapted to drought ending up a small increase in cassava yield in marginal regions which could easily lead to an increase in global production. Thus, to better understand the genetic and physiological traits of the drought-tolerant cultivars under water stress could provide fundamental knowledge for the genetic improvement of cassava for drought tolerance.

Currently, significant progress has been made toward understanding the mechanisms of cassava drought tolerance. The increasing volume of genomic resources for cassava is being enhanced and available to molecular breeding programs^3^. Genomes of cassava cultivars and wild ancestors have been available that reveals extensive interspecific hybridization and genetic diversity^4–6^. Genetic improvement for drought adaptation in cassava is also being enhanced by characterizing the crucial genes of the plant’s responding factors to abiotic stress, i.e. ethylene response factor family genes^7^, aquaporin family genes^8^, TCP transcription factors^9^, the mitogen-activated protein kinase kinase kinases gene family^10^, calcium sensors^11^, the KT/HAK/KUP family^12^, the late embryogenesis abundant protein family^13^.

Drought stress affects the growth, the morphological structure, photosynthetic characteristics, physiological and biochemical characteristics of plants^14–17^. Limited soil water can result in stomatal closure, which sequentially decreases CO_2_ intake and net photosynthesis^14,18–20^, finally resulting in reduced growth. Drought activates multiple responses such as variations in gene expression, productions of specific proteins and the high levels of metabolites^21^. Drought stress results in the accumulation of reactive oxygen species (ROS)^22^ due to its high reactivity as well as its toxicity^23^ can affect the biomembrane structure. Malondialdehyde (MDA) is the product of lipid peroxidation and has been extensively measured as an indicator for oxidative damage^24^. Plants adopt numerous adaptive approaches in response to drought stress, comprising escape, evasion and tolerance mechanisms, and among these strategies, one of the best is antioxidant enzymes production. The plant cells trigger antioxidant enzymes including superoxide dismutase (SOD), peroxidase (POD) and catalase (CAT) in order to scavenge the ROS excessively^25^. When membrane integrity was disrupted by stress, vital solutes will emerge from the organelles^26^, consequently resulting in electrolyte leakage (EL).

Proteomics methods have been recently applied to analyze proteins associated with cassava responses to drought stress and proteome analysis of leaves and roots of two cultivars (a drought-tolerant cultivar SC124 vs. a drought-sensitive cultivar Arg 7) at the seedling stage was operated after drought treatment^27^. The drought-response evidence of SC124 was acting like a ‘survival’ mode as early stomatal closure and a reduction in the levels of various photosynthetic proteins and photosynthetic capacity^27^. However, plant reactions in response to drought are enormously complicated and varied among plant species and growth stages, accompanied by water restriction periods^28–32^. It has been proved that there was a positive correlation between the total biomass and the root biomass and water stress at any time during the seedling stage reduces significantly the development of the cassava young shoots, ending up a limited root yield^33^. Another critical period for drought tolerance was in five to six months after planting and considered as the root bulking period^3^. Cassava was grown on many slopes or sandy soils that are prone to long-term droughts. Previous reports related to cassava drought tolerance mainly focused on the seedling stage only and there is still a lack of information of the physiological and molecular basis of cassava growth in response to long-term drought stress.

Thus, in the current study, the physiological and proteomic analysis of the drought-tolerance cassava cultivar XX048 at the tuberous root bulking stage were examined after a five-month drought treatment. The objectives of this study were to screen for proteins that are differentially expressed under long-term drought stress and to investigate the physiological and molecular basis of cassava growth in response to long-term drought stress. These results will provide deep understanding regarding mechanisms of cassava tolerance to long-term drought stress as well a new clue to cassava breeding.

## Results

### Effects of drought stress on the morphology of cassava

**Figure 1:**
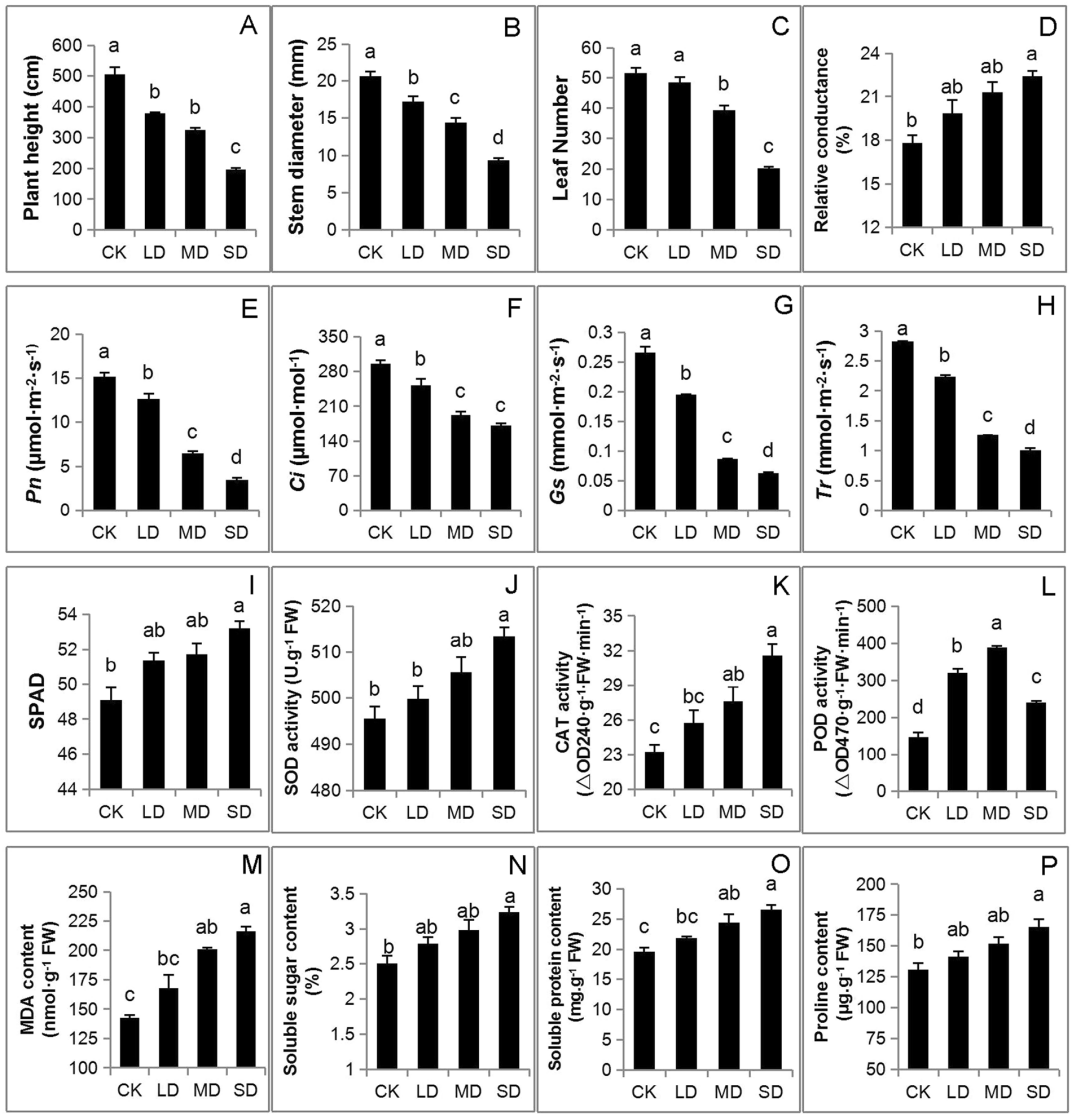
Morpho-physiological traits of cassava under drought stress. The treatments CK, LD, MD and SD indicate cassava exposed to RSWC as 80 ± 5%, 65 ± 5%, 75–85%, 50 ± 5% and 35 ± 5% for 5 months, respectively. The values are presented as means ± standard deviation (SD) of three independent biological repeats. The letters above the histogram indicate the statistical significance at the level of 0.05 (p < 0.05).

The morphological and physiological responses of the drought-tolerant cultivar XX048, against light (LD), moderate (MD) and severe (SD) drought stress levels were investigated with 6-month old plants exposed to different drought treatments for five months within the greenhouse. The well-watered plants were used as control (CK). Cassava plant height (Fig. 1A), stem diameter (Fig. 1B) and the number of green leaves (Fig. 1C) under drought stress were negatively influenced and decreased significantly compared with control.

### Changes of the photosynthesis parameters caused by long-term drought stress

Variations of the photosynthesis parameters of cassava leaves under drought treatment were also investigated and net photosynthesis (*Pn*), stomatal conductance (*Gs*), intercellular CO_2_ concentration (*Ci*) and leaf transpiration rate (*Tr*) were significantly influenced with the increased drought intensity (Fig. 1D–G). The results indicated that limited soil water led to stomatal closure, which sequentially decreases CO_2_ intake and net photosynthesis. Conversely, the leaf chlorophyll was positively influenced by drought and the SPAD value was increased by 8.38% under SD treatment compared with the control (Fig. 1H).

### Effects of drought stress on water content and cell membrane permeability of cassava leaves

As the intensity of drought increased, contents of the total water and the free water of cassava leaves reduced while the bound water content increased, which ended up a significant reduction of the ratio of free water and bound water (Table 1). Comparing to the control, the relative conductivity of the leaves was decreased by 12%, 19% and 26% under LD, MD and SD treatments after five months, respectively (Fig. 1I).

**Table 1:** Change of leaf free water\bound water content in leaves of cassava under drought stress.

Treatment	Total water content (%)	Free water content (%)	Bound water content (%)	Free water/bound water
CK	71.23 ± 0.27 a	53.12 ± 0.50 a	18.10 ± 0.27 c	2.93 ± 0.03 a
LD	70.28 ± 0.25 a	50.99 ± 0.62 ab	19.28 ± 0.25 bc	2.64 ± 0.03 b
MD	69.70 ± 0.32 ab	49.24 ± 0.28 b	20.46 ± 0.32 ab	2.41 ± 0.01 c
SD	68.30 ± 0.21 b	47.10 ± 1.01 b	21.20 ± 0.21 a	2.22 ± 0.05 d

The data are the mean value ± standard deviation (SD) of three independent biological repeats. The figures followed by different letters within the same column indicate the statistical significant at the level of 0.05 (p < 0.05).

### Effects of drought stress on contents of soluble sugar, soluble protein and free proline of cassava leaves

The contents of soluble sugar, soluble protein and free proline of cassava leaves were also positively influenced by drought and increased by 11–29%, 11–35% and 8–26% in the leaves from the light to the severe drought stress, respectively (Fig. 1J–L). Accumulation of these efficient osmolytes can lead to a low water potential of cells for resisting the drought stress^34^.

### Effects of drought stress on antioxidant enzyme activity

To cope with abiotic stress, plants accelerate the activities of antioxidant enzymes for the removal of ROS which can oxidize cellular components like proteins and lipids, DNA and RNA. The activities of the antioxidant enzymes SOD, POD and CAT of cassava leaves were also influenced by drought (Fig. 1M–O). The main function of SOD is to erase O^2−^ to form H_2_O_2_ and then CAT is followed to catalyze the decomposition of H_2_O_2_ into O_2_ and H_2_O. Therefore SOD and CAT activities were increased within a certain level of drought to sustain the stability of active oxygen while the intensity of drought increased (Fig. 1M,O). There were significant differences in POD activities among all the treatments (Fig. 1N). The MDA levels were increased by 41% and 51% at under MD and SD, respectively in comparison to the control (Fig. 1P).

### Correlation of the Morpho-physiological parameters of cassava

The correlation analysis showed that the plant height, stem diameter, number of green leaves, free water content, FW/BW, *Pn*, *Ci*, *Gs*, *Tr* were positively correlated with soil water content. The plant height, *Pn*, *Ci*, *Gs* and *Tr* were significantly positively correlated with soil water content. The stem diameter, free water content and FW/BW were extremely significantly positively correlated with the water content of soil. SPAD and CAT activity were significantly negatively correlated with soil water content. The bound water, cell membrane permeability, MDA content, soluble sugar content, soluble protein content, proline content and SOD activity were extremely negatively correlated with soil water content (Table S1).

### Identification of differentially expressed proteins in response to drought stress

**Figure 2:**
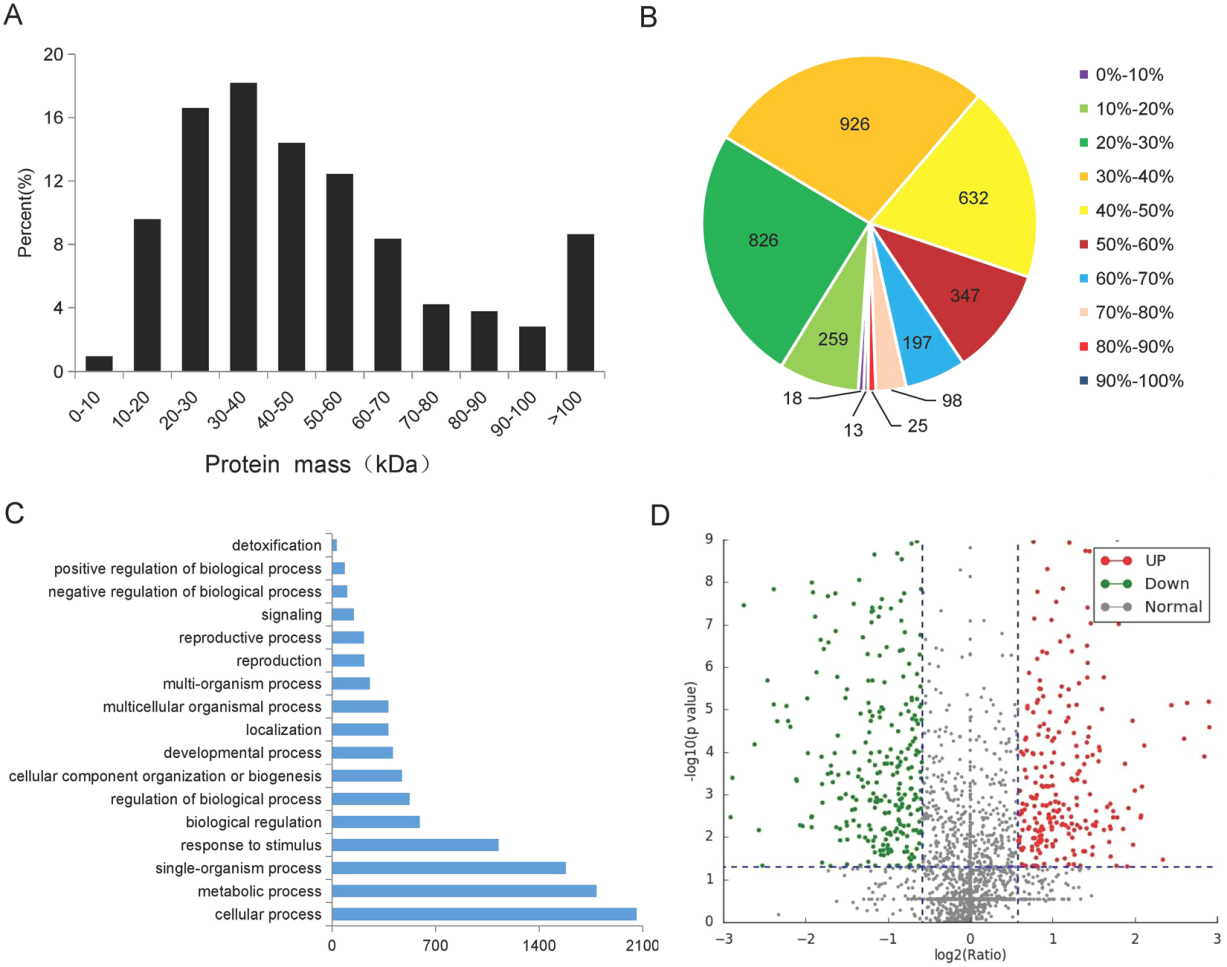
General information of the identified proteins. (**A**) The distribution of protein mass; (**B**) The distribution of the sequences coverage of the identified proteins; (**C**) Biological processes of the identified proteins; (**D**) The distribution of the relative expression of the quantified protein. The green and the red dots mean down-regulated and up-regulated differentially expressed proteins, respectively. The gray dots mean normal proteins.

On the basis of the physiological responses to water deficit conditions, samples of CK and the SD treatments were selected for profiling the proteome changes responded to drought. TMT labeling and HPLC fractionation was implemented, followed by high-resolution LC-MS/MS analysis and quantitative global proteome analysis. All the mass spectrometry data have been deposited into the iProX (http://www.iprox.org) with the identifier IPX0001316000. A total of 216 179 spectra were acquired and 177 687 of them matched to the reference spectra. There were 78 618 peptides were identified including 71 613 unique peptides. In total, 3 339 proteins (Table S2) were identified and their general information was shown in Fig. 2. Among these proteins, 352 of them between 0 to 20 kDa, 2060 of them were between 20 to 60 kDa, 640 proteins between 60 to 100 kDa and 289 proteins over 100 kDa (Fig. 2A). The peptide sequence coverage of most the identified proteins ranged from 20% to 60% (Fig. 2B). In total, 3270 identified proteins were quantified and related to 17 biological processes, including cellular process (61.6% of total), metabolic process (53.5%), single-organism process (47.3%) and response to stimulus (33.7%) (Fig. 2C).

**Figure 3:**
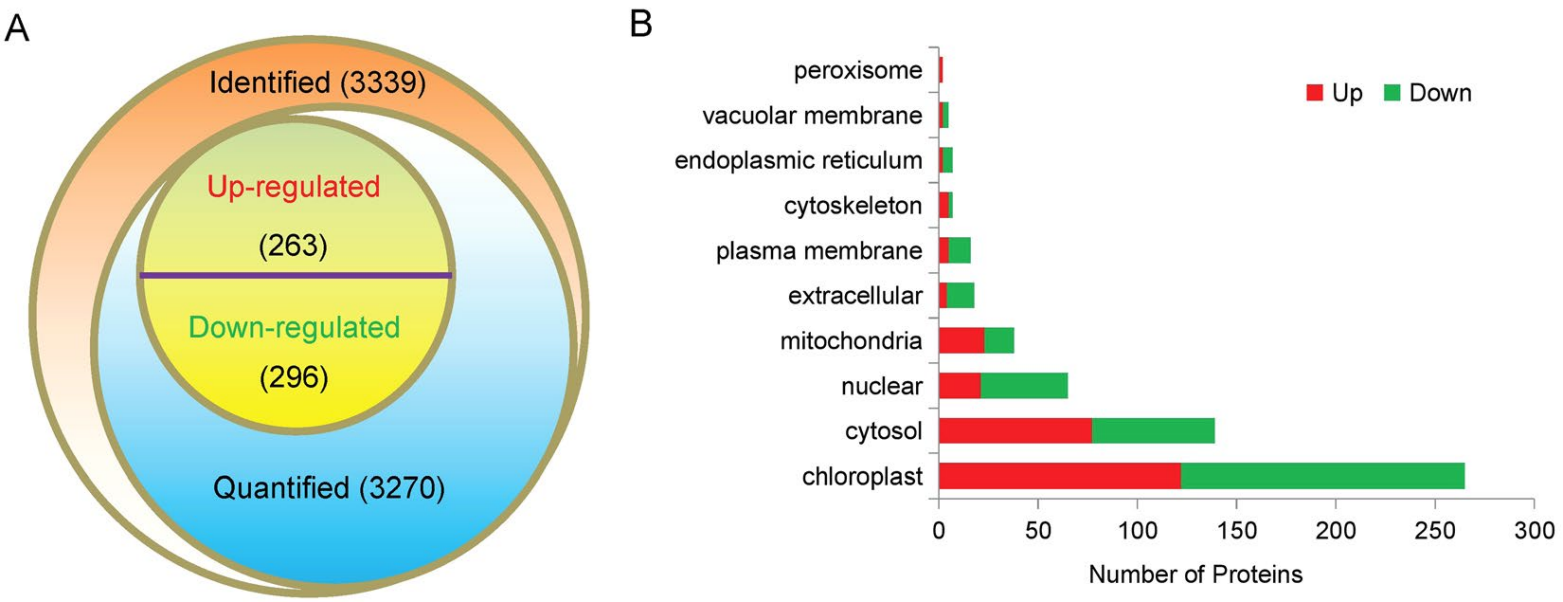
The number of the differentially expressed proteins in cassava leaves between the severe drought and the control treatments (**A**) and their subcellular localization (**B**). The red and the green bars in B represent the up-regulated and the down-regulated proteins, respectively.

In current study, 558 differentially expressed proteins (DEPs) performed dynamic changes between the severe drought treatment and the control with 262 DEPs up-regulated (SD: CK ratio ≥ 1.5, p-value < 0.05) and 296 DEPs down-regulated (SD: CK ratio ≤ 1/1.5, p-value < 0.05) (Figs 2D, 3A and Table S3). The subcellular localization of DEPs was predicted using WoLFPSORT prediction. Results indicated that most of the DEPs were localized in the chloroplast, cytosol, nuclear and mitochondria (Fig. 3B).

### GO functional classification and GO enrichment-based clustering analysis of DEPs

GO functional classification analysis was performed to reveal the function and the features of the DEPs using 3 sets of ontologies: biological process, cellular component and molecular function (Fig. S1). The biological process analysis showed that the top three processes were cellular process, metabolic process and single-organism process. For the cellular components category, most of the DEPs belonged to cell and cell part followed by organelle. The largest group in terms of molecular function was proteins with catalytic activity and the second largest group in this term was composed of binding proteins.

To better understand the preferred functional characteristics for the DEPs in response to long-term drought stress, GO enrichment based on clustering analyses was performed by dividing all quantified proteins into four quantiles according to the quantitative ratio in this study: Q1 (0–0.67), Q2 (0.67–0.83), Q3 (1.2–1.5) and Q4 ( >1.5). In the biological process category, the processes related to photosynthesis, translation and cellular response to abiotic stimulus were found to be significantly enriched in Q4, while the processes related to cell proliferation and RNA stabilization were highly enriched in Q1 (Fig. S2A). In the cellular component category, the up-regulated proteins were significantly enriched in the photosystem I reaction center, followed by chloroplast thylakoid membrane, while the down-regulated proteins were enriched in lytic vacuole and cell division site (Fig. S2B). Clustering analysis based on molecular function showed that the proteins with chlorophyll binding activity and oxidoreductase activity were enriched in Q4, and the proteins with the structural constituent of cytoskeleton and enzyme inhibitor activity were enriched in Q1 (Fig. S2C). Thus, these results indicate that the DEPs in response to long-term drought stress were involved in diverse biological processes and multifarious functions.

### Protein domain analysis of the DEPs

**Figure 4:**
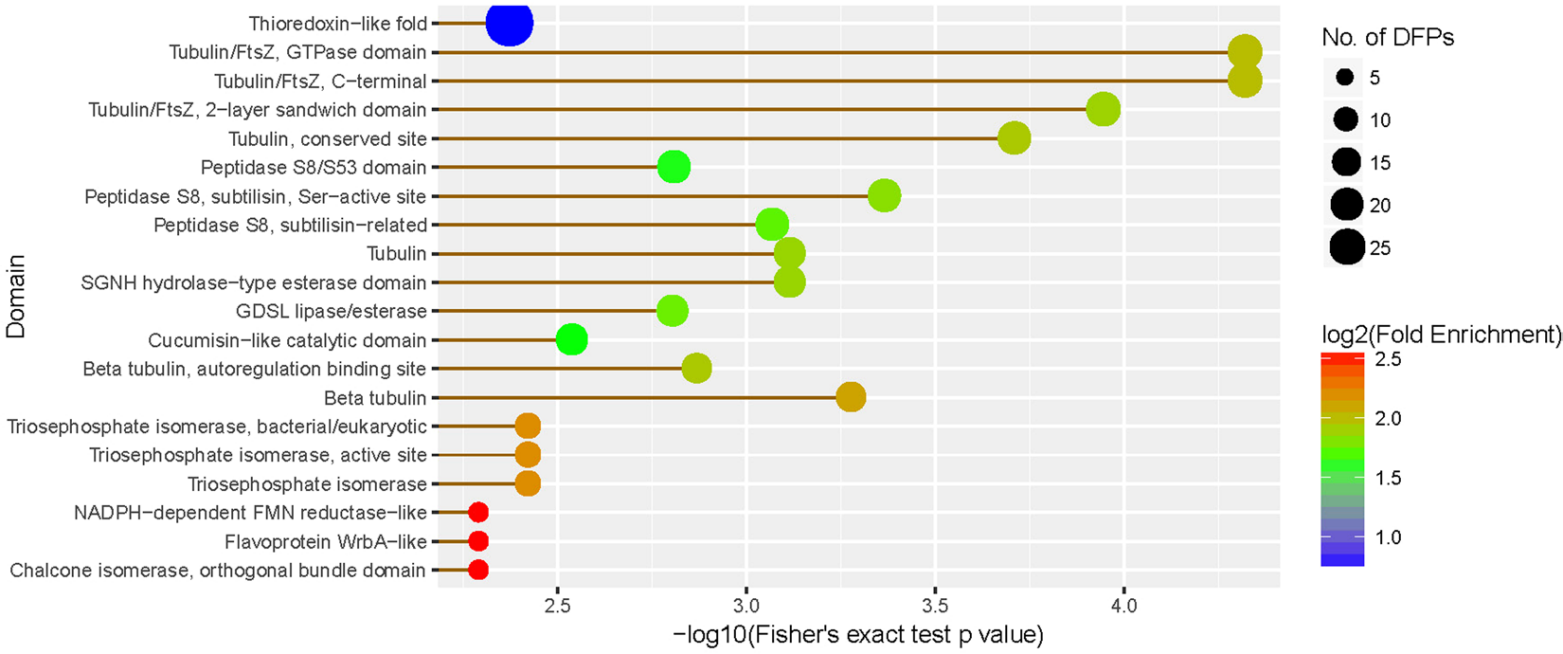
The domain identification of the differentially expressed proteins.

To address the domain features of the proteins altered by drought stress, domain annotation and enrichment analysis were performed. Results showed that tubulin/FtsZ domain and tubulin domain were highly enriched (Fig. 4). Further clustering analysis indicated that small heat shock protein HSP20, photosystem I Psa, transcription and translation associated domain were highly enriched in up-regulated proteins (Fig. S3). In the down-regulated proteins, various domains, such as tubulin/FtsZ, tubulin, GDSL lipase/esterase domain, ubiquilin and cell division protein FtsZ were found highly enriched (Fig. S3). These results indicated that the DEPs with many kinds of domain features were changed in response to drought stress.

### KEGG pathway analysis of the DEPs

**Figure 5:**
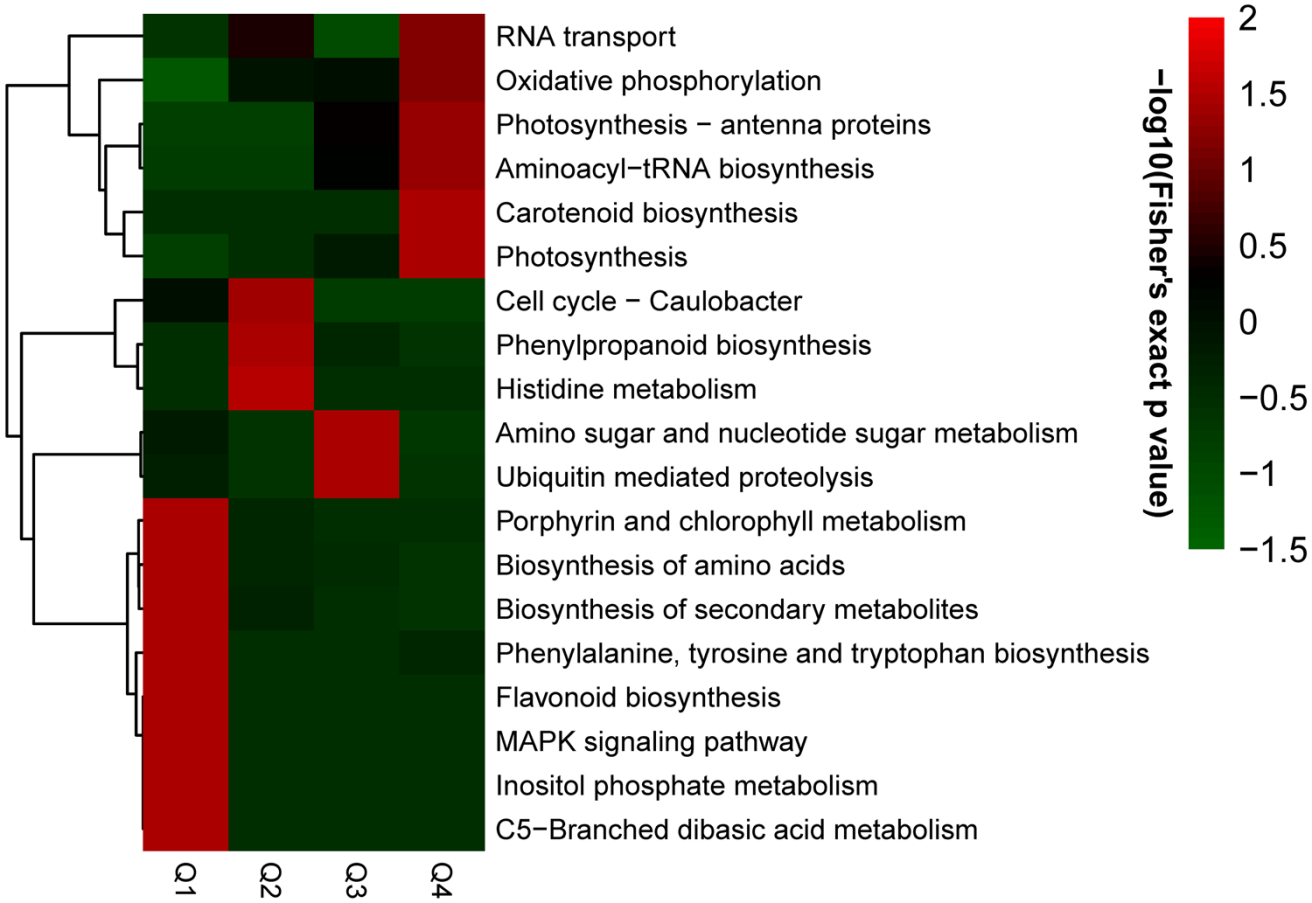
KEGG pathway based clustering analysis.

**Figure 6:**
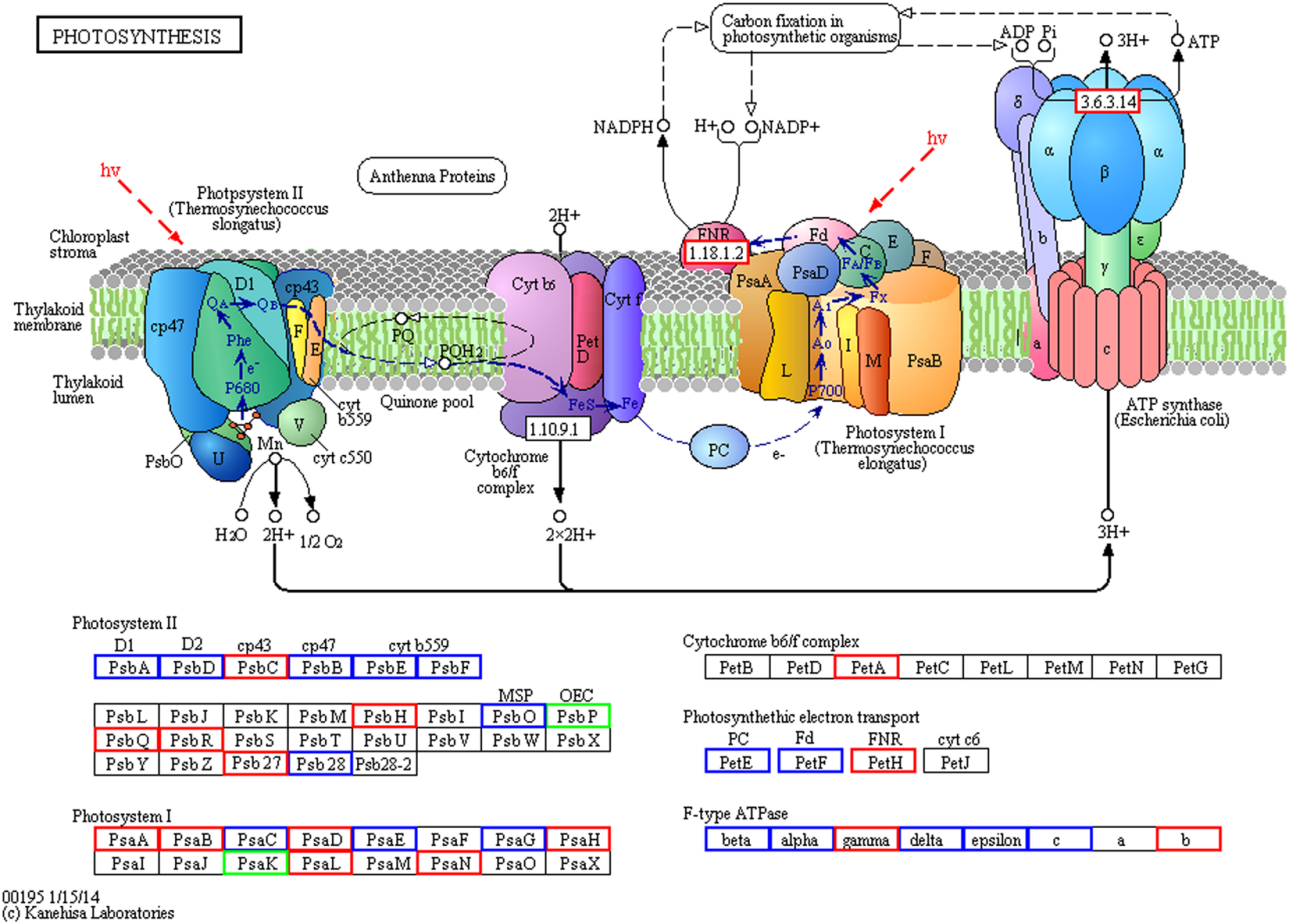
Changes of the differentially expressed proteins in photosynthesis pathway. The proteins in red and green are up-regulated and down-regulated expressed, respectively. The proteins in blue are expressed at background level. The pathway map was generated using KEEG^57–59^.

To identify pathways regulated by drought stress, KEGG pathway based on enrichment analysis of the DEPs was performed (Fig. 5). The up-regulated proteins were significantly enriched in photosynthesis (Fig. 6), carotenoid biosynthesis, Aminoacyl-tRNA biosynthesis, photosynthesis-antenna proteins, oxidative phosphorylation and RNA transport. The down-regulated proteins were significantly enriched in porphyrin and chlorophyll metabolism, C5-branched dibasic acid metabolism and MAPK signaling pathway. These results indicate that the DEPs were highly associated with photosynthesis.

### Protein-protein interaction (PPI) network analysis of DEPs

**Figure 7:**
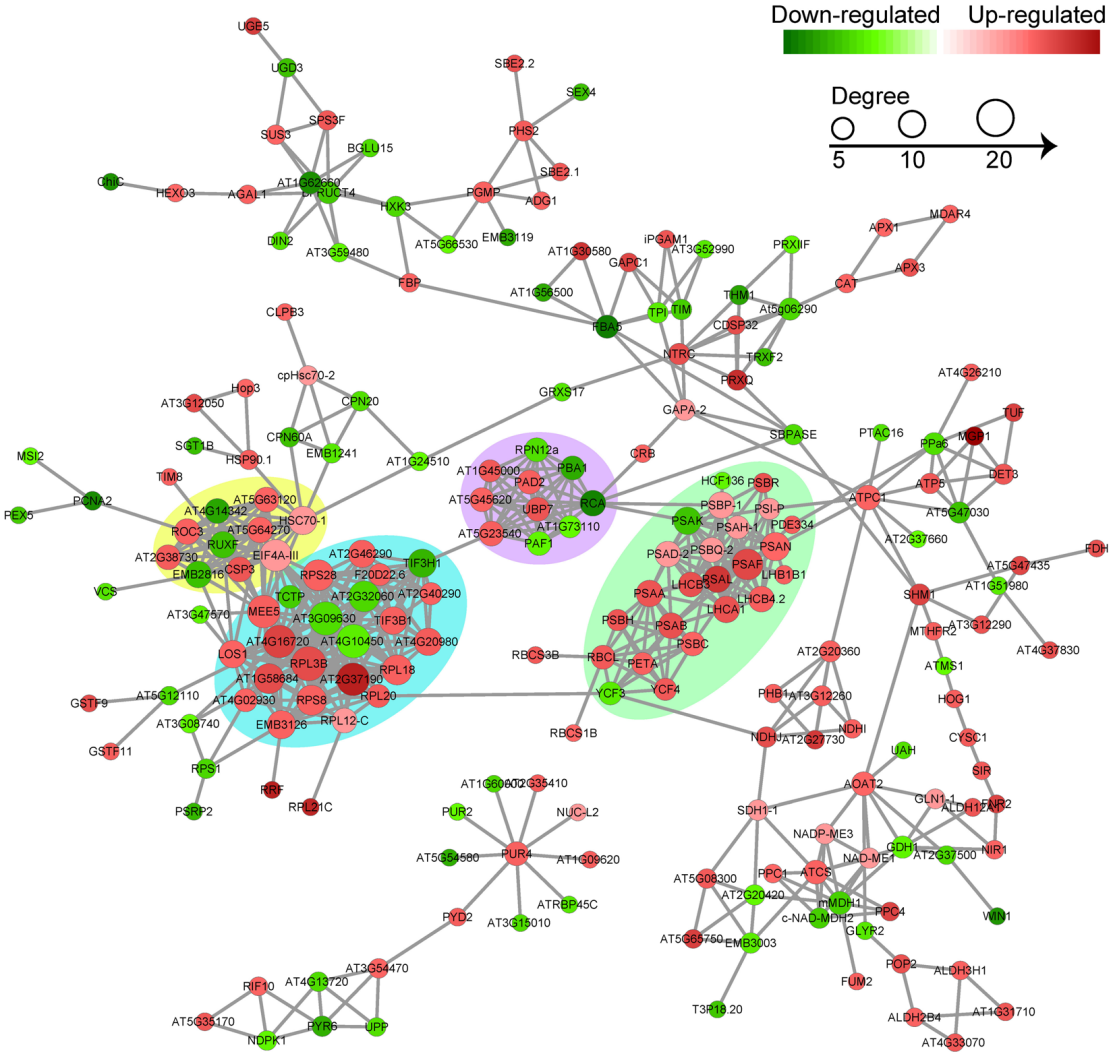
Protein-protein interaction network analysis of drought stress-responsive DEPs. DEPs related to photosynthesis, protein synthesis, transcription and proteasome were indicated in backgrounds of green, blue, yellow and purple, respectively.

To further understand the influence of drought stress on protein interactions, we searched for DEPs against the String database, and protein-protein interaction network was constructed. Since there is still no PPI data of cassava proteins available in the String-database, the *Arabidopsis thaliana* was used as the reference to get homologs proteins (Table S4). The global network graph of these interactions was shown in Fig. 7 and many proteins were involved in multiple interactions. Apparently, four protein-protein interaction networks which related to photosynthesis, protein synthesis, transcription and proteasome networks were significantly enriched and more up-regulated proteins were involved into these networks than the down-regulated ones.

### The correlation of expression levels between mRNA and proteins

**Figure 8:**
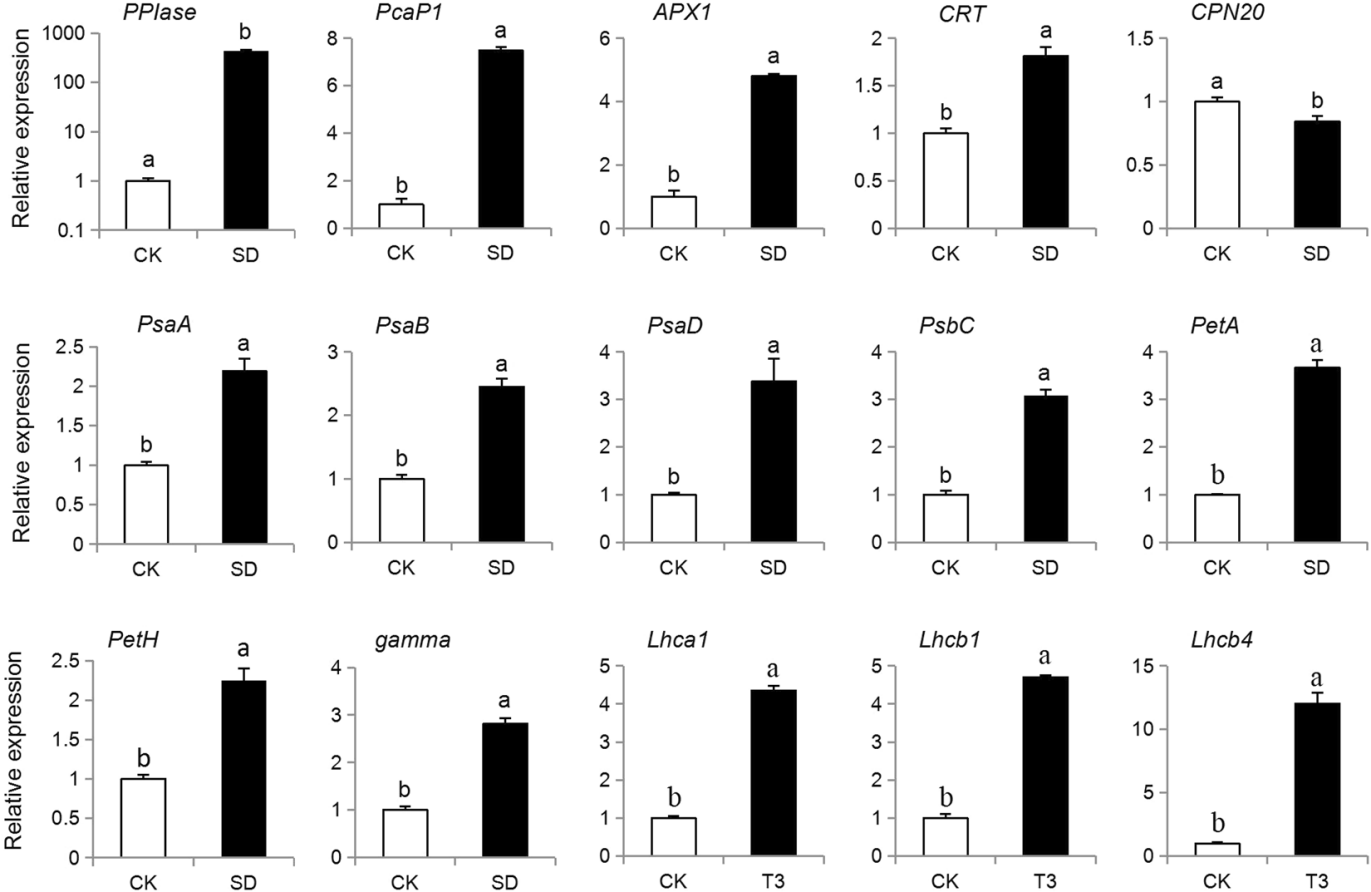
Relative expression of 15 genes at mRNA level.

In order to assess the correlation of expression levels between mRNA and protein, qPCR was applied. Fifteen genes (Table S5) which regulated to the DEPs (five genes correlated to abiotic stress and the rest were linked to photosynthesis) were chosen and the relative expressions were shown in Fig. 8. The expressions of the *PPIase*, *PCaP1*, *APX1*, *CPN20*, *PsaA*, *PsaB*, *PsaD*, *PsaC*, *PetA*, *PetH*, *gamma*, *Lhca1*, *Lhcb1* and *Lhcb4* were consistent with the corresponding proteins, which indicated that expressions of these proteins were regulated at the transcriptional level.

## Discussion

To better understand physiological and proteomic mechanisms of cassava resistant to long-term drought stress and then revealing the main regulatory genes under drought stress could benefit for breeding drought-resistant varieties. According to previous studies, two drought resistance strategies of cassava were reported^27^. One way was showing as sensing and shedding of old leaves and leaving young leaves green and remained high photosynthesis capacity. The other way was acting as a ‘survival’ mode as early stomatal closure, leaf wilting, decreased photosynthesis and was more capable of surviving the extended severe drought than the previous strategy. Cassava cultivar XX048 was chosen as the examined materials because its strong ability to adapt to drought and a relatively high yield still could be achieved even under severe drought stress^35^. Thus, a five-month drought stress treatment was applied on well-watered cassava young shoots after one-month planting and it was acting as the ‘survival’ mode under drought stress.

In this report, the plant height and stem diameter were significantly decreased after the long-term drought stress. This should be due to the inhibition of cell growth and differentiation resulting in slow apical meristem and development. Cell proliferation and growth are significant throughout the life cycle of plants. In the present study, 15 out of 16 proteins (Table S3) related to cell growth and division were down-regulated, indicating that the drought environment affected the proliferation and growth of cassava cells, causing the decrease of cassava plant height, stem diameter and the number of green leaves. As the intensity of drought increased, the total water, free water and the ratio of free water and bound water levels reduced in the leaves of cassava, while the bound water content increased. This may be due to the water content in different states are closely linked with plant growth and resistance. When the ratio of free water content to bound water content was high, the metabolic activity of plant tissue increased^36^. The data were consistent with Zhang *et al*.^37^ who also reported that increasing drought stress increased bound water content in pepper leaves. Thus, long-term drought treatment evidently suppressed the normal growth and reduced the metabolic activity of cassava.

Photosynthesis parameters, including *Pn*, *Ci*, *Gs* and *Tr* were significantly decreased with the increase of the drought intensity, which indicated that limited soil water led to the stomatal closure of cassava leaves, which sequentially decreased CO_2_ intake and then lead to a low net photosynthesis. Similar results were observed in a drought-tolerant apple cultivar ‘Qinguan’ responses to drought, but its *Pn* was still higher than water-sensitive apple cultivar ‘Naganofuyi No.2’ under the same water condition^38^. When taking the investigation into the proteomic level, the results of the KEGG pathway analysis showed that the up-regulated proteins were significantly enriched in photosynthesis. This was consistent with the proteomic analyzed results of the tolerant-drought apple cultivar ‘Qinguan’ responses to drought, also demonstrating that regulation of photosynthesis process plays the most key role under drought.

Photosynthesis is composed of two steps, namely, photoreaction (including primary reaction, electron transport and photophosphorylation) and dark reaction. Light-harvesting chlorophyll a/b binding proteins play important roles in maintaining the thylakoid membrane structure, adjusting the distribution of excitation energy between PS I and PS II, light protection and adaptation to the variety of environments. In the present study, the up-regulated chlorophyll a/b binding proteins not only maintained chlorophyll biosynthesis and photosystem stability under drought condition, but also led to improving non-photochemical quenching, dissipating excess excitation energy, protecting the photosynthetic electron transport chain, and maintaining the reaction center activation^38^. In this study, the physiological analysis revealed that the SPAD value was increased under drought stress (Fig. 1H) may due to the change of chlorophyll a/b binding proteins. The increase of PSII and PSI subunits were related to photosystem repair, which would protect the reaction centers from actions by stromal proteases and modulate the electron transfer efficiency. In the present study, many PSII and PSI subunits were increased, which was beneficial to photosystem repair and protecting the reaction centers, thus kept photosynthetic electron transport chain function. The PSII is particularly vulnerable and its reaction center requires rebuilding^39^. When the rate of damage exceeds that of repair, the photosynthetic electron transport chain is limited, and leads to photoinhibition. Under drought, the rates of both damage and repair can be rapid, thus a high level of PSII activity is maintained, for example, by increasing of the CP43 and R subunits, which were increased in drought treated cassava leaves, helping photosynthetic electron transport chain to keep smooth in cassava leaves. Similar data have also been reported by Hui Liu^40^, which proposed that the photosynthetic electron transport chain was greatly hampered during water stress resulting in excess excitation energy when the rate of damage exceeds that of repair. This phenomenon increased the production of ROS, possibly revealing the strategy of plant to actively cope with drought stress. Ribulose bisphosphate carboxylase (Rubisco) is an important carboxylase in the dark reaction, as well as essential oxygenase in photorespiration which is a key enzyme in assessing the rate of carbon assimilation in photosynthesis^41^. Sedoheptulose-1,7-bisphosphatase (SBPase) activates the supply of RuBP to Rubisco, thereby enhancing photosynthesis efficiency under stress. In the present study, Rubisco and phosphoenolpyruvate carboxylase were up-regulated, while fructose-bisphosphate aldolase and SBPase were down-regulated, indicating that the regeneration of RuBP was affected by drought stress, thus affecting carbon assimilation.

Stress often causes protein denaturation. Therefore, the balance between synthesis and degradation of proteins is beneficial to maintain the cellular normal metabolic activities^42^. There were 43 DEPs involved in protein synthesis (Table S3). Ribosomal proteins played key role in stress response and cell viability. Ribosome overexpression can enhance the stress resistance of plants^43^. The abundance of ten ribosomal proteins (Table S3) increased, whereas six decreased in the present study. Wu Li *et al*.^44^ stated that levels of the ribosomal proteins hampered while some specific ribosomal components improved when subjected to drought stress. Furthermore, the results in the present study indicated that 8 out of 9 eukaryotic translation initiation factor 5 A (eIF5A, Table S3) increased under drought stress. Previous research reported that eIF5A plays a crucial role in plants responding to external stresses^45–47^. The abundance of elongation factors increased in the present study. The differential regulation of different components of the translation machinery shows complicated regulation mechanisms. This governs protein synthesis to adopt to drought stress in plants.

Suitable protein folding and processing is essential in order to maintain normal cellular function during harsh environmental conditions. And the heat shock proteins participate in protein folding and degradation during cell metabolism^48^ and help plant to protect it against deteriorating environmental conditions through restoring normal protein conformation^49^. In the current study, expressions of seven heat shock proteins were found (Table S3) increased while other six heat shock proteins were decreased. Furthermore, peptidyl-prolyl cis-trans isomerase (PPIase) increased by 6.22-fold under long-term drought stress. These findings suggest that PPIase may play a vital role in resisting against drought of cassava.

Ubiquitin/26 S proteasome pathway (UPP) is one of the most effective and highly selective protein degradation pathways. There were 6 DEPs related to UPP increased and 6 decreased under drought stress condition. These findings suggest that complex changes of UPP happened in leaves under long-term drought stress.

In total, there were 14 proteins were identified in this experiment related to stress and defense (Table S3). The increases of CAT and Fe-SOD were consistent with the physiological data. The APX (ascorbate peroxidase) was increased in the present study. APX was an important component of the AsA-GSH redox pathway, and a key enzyme to remove H_2_O_2_ (especially H_2_O_2_ in chloroplasts)^50^.

Tubulin is a main driver of the cytoskeleton which is important for maintenance of cell morphology, cell division, signal transduction and so on. The change of environment can affect the expression of plants tubulin. The present study drew similar results that tubulin chains (Table S3) were down-regulated under drought stressed environment. Actin is a highly conserved protein and exists in all eukaryotic cells. It is involved in cell division, movement, migration, morphological maintenance, growth and other important physiological activities^51^. In the present study, the expressions of actin were down-regulated under long-term drought condition. Thus, the cellular structure of cassava can be severely damaged by the drought stress while the stress increased to a certain level.

Furthermore, the expressions of 14 out of 15 genes were consistent with their corresponding proteins, which indicated that the correlation between the transcript levels of genes and their protein abundance was strong in the present study.

In addition, there were many DEPs involved with TCA, metabolism, transcription, cell membrane transport, and signal transduction under long-term drought treated cassava leaves. These data will provide better understanding regarding the complexity of leaf protein variations that occur under drought stress. These data will be useful for further functional research on each identified protein. These results substantially improved the knowledge regarding molecular mechanisms involved in drought stress resistance of cassava crops.

## Materials and Methods

### Materials

Cassava cultivar ‘Xinxuan 048’ (XX048) was bred by Guangxi University (GXU) and it is one of the most popular varieties in China because of its high root yield and its higher adaptabilities to drought stress than other cultivars^52^. Thus, XX048 was used in this study as a drought-resistant cassava model.

### Experimental design and operations of the experiment

The drought-stress treatment was set up as three levels, including the relative soil water content (RSWC) as 65 ± 5% (light drought stress, LD) of the maximum soil water holding capacity (MSWHC), RSWC as 50 ± 5% (moderate drought stress, MD) of MSWHC and RSWC as 35 ± 5% (severe drought stress, SD) of MSWHC. The well-watered treatment (RSWC as 80 ± 5% of MSWHC, CK) was applied as the control.

The experiment was operated in a greenhouse in GXU in 2015. In total, about 800 kg topsoil (0–20 cm) was collected from a farm, air-dried and well mixed before the experiment. The characteristics of the soil were analyzed and the results were shown as pH = 6.6, and the contents of organic matter, available N, P, K were 23.38 mg.kg^−1^, 53.23 g.kg^−1^, 77.57 g.kg^−1^ and 6.30 g.kg^−1^, respectively. Before filling the soil, three holes were made in each polyethene pot (39 cm × 58 cm × 40 cm) in order to avoid water accumulation at the bottom. About 5 kg of sand was paved at the bottom of the pot and then filled with 66 kg of the air-dried soil on top. In total, twelve plots, three replicates for each treatment, were prepared. Two stem segments were planted in each plot and one shoot was kept for each plant.

After well-water the plants 30 days, the drought treatment was then followed. To maintain the soil water condition, the soil sample was collected from 0–25 cm depth from each plot during the every-third-day and the moisture content was analyzed. The watering plan was adjusted according to the moisture content of the soil. The plants were contentiously watered to maintain the corresponding water content. These treatments continued for five months.

### Sampling and processing

Cassava leaf sample was collected on 190 days after planting during the root tuber expansion stage. The fourth and the fifth leaves which were fully expanded were chosen and the sample was immediately frozen in liquid nitrogen, stored at −80 °C for further analysis. Other fresh leaves were also sampled and used to assess cell membrane permeability and free water and bound water contents.

### Physiological parameters

Photosynthesis parameters, including *Pn*, *Ci*, *Gs* and *Tr*, were measured using photosynthesis LI-6400XT (Li-COR, Lincoln, NE, USA) at 10:00–11:00 am on 189 DAP, one day before the sampling. Chlorophyll content was also measured on that day using relative chlorophyll meter (SPAD-502 plus, TOP Instrument, China).

The total water content of cassava leaf was measured by the oven-dry method and the bound water of cassava leaf was measured using Abbe refractometer (WZS-1, Shanghai optical instrument factory, China). The free water content was then obtained by subtracting the bound water content from the total water content according to Zou^36^. Cell membrane permeability was analyzed according to Zhou *et al*.^53^.

Antioxidants activities of cassava leaf, including the activities of SOD, POD and CAT and the content of MDA were measured^54^. The osmotic adjustment substances in cassava leaf, i.e. soluble sugar content, soluble protein and proline content were analyzed^54^.

### Proteomic analysis

Only two samples from the control and the severe drought stress treatment were chosen for the proteomic analysis. The process of total protein extraction, trypsin digestion, tandem mass tag (TMT) labeling, HPLC fractionation and the LC-MS/MS analysis were conducted according to Gao *et al*.^55^.

### QRT-PCR analysis

The samples from the control and the severe drought stress treatment were also chosen for qRT-PCR analysis. Total RNA was extracted from the sample by RNA extraction kit (Huayang), cDNA was reversed transcribed from 1 μg total RNA using cDNA Synthesis Kit (Takara) and primers were designed using primer 5.0 (Table S5). The cassava Ribosomal protein L7Ae (*rpl7Ae*) gene was used as an endogenous control for normalization. The reaction system for PCR contained 5 μl 2 × ChamQ SYBR qPCR Master mix (Vazyme), 1 μl template cDNA and 0.5 μl of the primers by adding ddH_2_O up to 10 mL. The operating process was set as the following protocol: 95 °C for 3 min, followed by 50 cycles of 95 °C for 10 s, 50 °C–60 °C (varied according to different primers) for 10 s and 72 °C for 15 s. The reactions were performed with three replications. The relative gene expression levels were calculated using the 2^−ΔΔCT^ method^56^.

### Bioinformatics and data analysis

The proteins were identified using Sequest software integration in Proteome Discoverer (version 1.3, Thermo Scientific) with Uniprot *Manihot esculenta* database (38,254 items, up-data in August 2018) according to the LC-MS/MS data. Trypsin was chosen as enzyme and two missed cleavages were allowed. Carbamidomethylation (C) was set as a fixed modification. Oxidation (M) and acetylation in N-Term was set as variable modification. The searches were performed using a peptide mass tolerance of 20 ppm and a production tolerance of 0.05 Da, resulting in a 5% false discovery rate (FDR).

Gene Ontology (GO) annotation proteome was determined within the UniProt-GOA database (http://www.ebi.ac.uk/GOA/). InterProScan was used for protein domain annotation based on protein sequence alignment method and the InterPro (http://www.ebi.ac.uk/interpro/) domain database. The annotation of the protein pathway was done within the Kyoto Encyclopedia of Genes and Genomes database (KEGG, https://www.kegg.jp/)^57–59^. The protein-protein interaction (PPI) network of the differentially expressed proteins (DEPs) was constructed within the String database (https://string-db.org/, Accessed August 20, 2018). Since there was no data available for cassava, the *Arabidopsis thaliana* was adopted as reference. The PPI networks were then constructed using String software with Confidence Scores greater than 0.9^60^.

Statistical analysis was conducted using ANOVA, which was performed by using SPSS 18.0 (SPSS Science, Chicago, IL, USA) to Duncan’s tests. A value of *P* < 0.05 was considered a statistically significant difference.

## Electronic supplementary material

Supplementary Figures

Supplementary Tables

## References

[1] Xie H (2016). iTRAQ-based quantitative proteomic analysis reveals proteomic changes in leaves of cultivated tobacco (Nicotiana tabacum) in response to drought stress. Biochemical & Biophysical Research Communications..

[2] Ipcc, W. G. I. Climate change 2007: impacts, adaptation and vulnerability. Working Group II contribution to the Intergovernmental Panel on Climate Change. Fourth assessment report. Summary for policymakers (2007).

[3] Okogbenin E (2013). Phenotypic approaches to drought in cassava: review. Front Physiol..

[4] Prochnik S (2012). The cassava genome: current progress, future directions. Trop Plant Biol..

[5] Wang W (2014). Cassava genome from a wild ancestor to cultivated varieties. Nat Commun..

[6] Egesi C (2016). Sequencing wild and cultivated cassava and related species reveals extensive interspecific hybridization and genetic diversity. Nat Biotechnol..

[7] Fan W (2016). The ERF transcription factor family in cassava: genome-wide characterization and expression analyses against drought stress. Sci Rep..

[8] Putpeerawit P, Sojikul P, Thitamadee S, Narangajavana J (2017). Genome-wide analysis of aquaporin gene family and their responses to water-deficit stress conditions in cassava. Plant Physiol Biochem..

[9] Lei N (2017). Phylogeny and expression pattern analysis of TCP transcription factors in cassava seedlings exposed to cold and/or drought stress. Sci Rep-Uk..

[10] Ye J (2017). The MAPKKK gene family in cassava: Genome-wide identification and expression analysis against drought stress. Sci Rep-Uk..

[11] Wei H (2018). Genome-wide analyses of calcium sensors reveal their Involvement in drought stress response and storage roots deterioration after harvest in cassava. Genes-Basel..

[12] Ou W (2018). Genome-wide identification and expression analysis of the KUP family under abiotic stress in cassava (*Manihot esculenta* Crantz). Front Physiol.

[13] Wu C (2018). The late embryogenesis abundant protein family in cassava (*Manihot esculenta* Crantz): Genome-wide characterization and expression during abiotic stress. Molecules.

[14] Elsharkaway MA, Cock JH (1984). Water use efficiency of cassava: effects of air humidity and water stress on stomatal conductance and gas exchange. Crop Sci..

[15] El-Sharkawy MA, Cock JH (1987). Response of cassava to water stress. Plant & Soil..

[16] Elsharkawy MA, Cadavid LF (2002). Response of cassava to prolonged water stress imposed at different stages of growth. Exp Agr..

[17] Ge Y (2014). Physiological and biochemical responses of Phoebe bournei seedlings to water stress and recovery. Acta Physiol Plant..

[18] Cayón MG, El-Sharkawy MA, Cadavid LF (1997). Leaf gas exchange of cassava as affected by quality of planting material and water stress. Photosynthetica..

[19] Lawlor DW, Tezara W (2009). Causes of decreased photosynthetic rate and metabolic capacity in water-deficient leaf cells: a critical evaluation of mechanisms and integration of processes. Ann Bot-London..

[20] Reddy AR, Chaitanya KV, Vivekanandan M (2004). Drought-induced responses of photosynthesis and antioxidant metabolism in higher plants. J Plant Physiol..

[21] Lokko Y (2007). Characterization of an 18,166 EST dataset for cassava (*Manihot esculenta* Crantz) enriched for drought-responsive genes. Plant Cell Rep..

[22] Mittler R (2002). Oxidative stress, antioxidants and stress tolerance. Trends Plant Sci..

[23] An Y, Liang Z (2013). Drought tolerance of Periploca sepium during seed germination: antioxidant defense and compatible solutes accumulation. Acta Physiol Plant..

[24] Blokhina, O., Virolainen, E. & Fagerstedt, K. V. Antioxidants, oxidative damage and oxygen deprivation stress: a review. Ann Bot-*London. 91 Spec No*, **179** (2003). 10.1093/aob/mcf118 PMC4244988 12509339

[25] Mh CDC (2008). Drought stress and reactive oxygen species: production, scavenging and signaling. Plant Signaling & Behavior..

[26] Valentovic P, Luxova M, Kolarovic L, Gasparikova O (2006). Effect of osmotic stress on compatible solutes content, membrane stability and water relations in two maize cultivars. Plant Soil & Environment..

[27] Zhao P (2015). Analysis of different strategies adapted by two cassava cultivars in response to drought stress: ensuring survival or continuing growth. J Exp Bot..

[28] Farooq M, Wahid A, Kobayashi N, Fujita D, Basra SMA (2009). Plant drought stress: effects, mechanisms and management. Agron Sustain Dev..

[29] Fahad, S. & Bano, A. Effect of salicylic acid on physiological and biochemical characterization of maize grown in saline area. *Pak J Bot*. **44**, (2012).

[30] Aslam, M., Zamir, S. I., Anjum, S. A., Khan, I. & Tanveer, M. An investigation into morphological and physiological approaches to screen maize (Zea mays L.) hybrids for drought tolerance. *Cereal Res Commun*. **1** (2014).

[31] Fahad S (2015). Crop plant hormones and environmental stress. Springer International Publishing..

[32] Saud S (2017). Effects of nitrogen supply on water stress and recovery mechanisms in Kentucky Bluegrass Plants. Front Plant Sci..

[33] Howeler, R., Lutaladio, N. & Thomas, G. Save and grow: cassava: a guide to sustainable production intensification. Food and Agriculture Organization of the United Nations (Rome 2013)

[34] Souza RP, Machado EC, Silva JAB, Lagôa AMMA, Silveira JAG (2004). Photosynthetic gas exchange, chlorophyll fluorescence and some associated metabolic changes in cowpea (Vigna unguiculata) during water stress and recovery. Environ Exp Bot..

[35] Wang, Z. P., Liu, H. B. & Luo, X. L. Water-requirement characteristics of two cassava varieties. *Journal of Southern Agriculture*. (2012).

[36] Zou, Q. *Experimental Guidance of Plant Physiology*. Chinese Agricultural Press. (Beijing 2000)

[37] Zhang DY (2010). Effect of water stress on some physiology indices of pepper leaves. Hubei Agricultural Sciences..

[38] Zhou S (2015). Physiological and proteome analysis suggest critical roles for the photosynthetic system for high water-use efficiency under drought stress in Malus. Plant Sci..

[39] Foyer CH, Shigeoka S (2010). Understanding Oxidative Stress and Antioxidant Functions to Enhance Photosynthesis. Plant Physiol..

[40] Liu H (2015). Physiological and comparative proteomic analysis reveals different drought responses in roots and leaves of drought-tolerant wild wheat (Triticum boeoticum). Plos One..

[41] Tingqi HE, Bingqiang XU, Guo A, Wang L (2013). Comperative Proteomic of Chloroplast From Different Species of *Manihot esculenta*. Chinese Journal of Tropical Crops..

[42] Reinbothe C, Pollmann S, Reinbothe S (2010). Singlet oxygen signaling links photosynthesis to translation and plant growth. Trends Plant Sci..

[43] Berberich T, Uebeler M, Feierabend J (2000). cDNA cloning of cytoplasmic ribosomal protein S7 of winter rye (Secale cereale) and its expression in low-temperature-treated leaves. Biochimica Et Biophysica Acta..

[44] Wu L (2015). Identification of early salt stress responsive proteins in seedling roots of upland cotton (Gossypium hirsutum L.) employing iTRAQ-based proteomic technique. Front Plant Sci..

[45] Zhang, L. J. *et al*. Cloning and expression analysis of the eukaryotic translation initiation factor 5A gene (PtoeIF5A4) in Populus tomentosa. *Journal of Agricultural Biotechnology*. (2013).

[46] Parkash J, Vaidya T, Kirti S, Dutt S (2014). Translation initiation factor 5A in Picrorhiza is up-regulated during leaf senescence and in response to abscisic acid. Gene..

[47] Xu J, Zhang B, Jiang C, Feng M (2011). RceIF5A, encoding an eukaryotic translation initiation factor 5A in Rosa chinensis, can enhance thermotolerance, oxidative and osmotic stress resistance of Arabidopsis thaliana. Plant Mol Biol..

[48] Neta-Sharir I, Weiss D (2005). Dual role for tomato heat shock protein 21: protecting photosystem II from oxidative stress and promoting color changes during fruit maturation. Plant Cell..

[49] Wang W, Vinocur B, Shoseyov O, Altman A (2004). Role of plant heat-shock proteins and molecular chaperones in the abiotic stress response. Trends Plant Sci..

[50] Najami N (2008). Ascorbate peroxidase gene family in tomato: its identification and characterization. Mol Genet Genomics..

[51] Tang, W. *Studies on the molecular mechanism of nuclear actin in gene transcription regulation*. Northeast Normal University. (Changchun 2009).

[52] Wang, Z. P., Liu, H. B. & Luo, X. L. Water-requirement characteristics of two cassava varieties. Journal of Southern Agriculture (2012).

[53] Zhou, Z. & Li, Z. *Experimental guidance of plant physiology*. Guangxi University. (Nanning 2005).

[54] Luo X, Huang Q (2012). Studies on the cold resistance of cassava. Journal of Agricultural Science..

[55] Gao X (2017). Proteomic analysis reveals large amounts of decomposition enzymes and major metabolic pathways involved in algicidal process ofTrametes versicolorF21a. Sci Rep..

[56] Livak KJ, Schmittgen TD (2001). Analysis of relative gene expression data using real-time quantitative PCR and the 2^−△△^ method. Methods..

[57] Kanehisa Furumichi M, Tanabe M, Sato Y, Morishima K (2017). KEGG: new perspectives on genomes, pathways, diseases and drugs. Nucleic. Acids. Res..

[58] Kanehisa M, Sato Y, Kawashima M, Furumichi M, Tanabe M (2016). KEGG as a reference resource for gene and protein annotation. Nucleic Acids Res..

[59] Kanehisa M, Goto S (2000). KEGG: Kyoto Encyclopedia of Genes and Genomes. Nucleic. Acids. Res..

[60] Franceschini A (2013). STRINGv9.1: protein-protein interaction networks, with increased coverage and integration. Nucleic Acids Res..

