# Supplementary material for: Physiological and proteomic analysis on long-term drought resistance of cassava (Manihot esculenta Crantz)
Source: Sci Rep. 2018 Dec 19;8:17982. doi: 10.1038/s41598-018-35711-x (PMC6299285; doi:10.1038/s41598-018-35711-x)
Supplement: Supplementary file 1 — Supplementary Figures [file 41598_2018_35711_MOESM1_ESM.doc]

**Physiological and** **proteomic analysis on long-term drought resistance of cassava (*Manihot esculenta* Crantz)**

**Zhongying Shan^1^, Maogui Wei^1^, Tangwei Huang^1^, Aziz Khan^1^, Yanmei Zhu^1^ & Xinglu Luo^1,2*^**

^1^College of Agronomy, Guangxi University, Nanning, 530004, China

^2^State Key Laboratory for Conservation and Utilization of Subtropical Agro-bioresources, Nanning, 530004, China

^*^Correspondence author: luoxinglu@sina.com


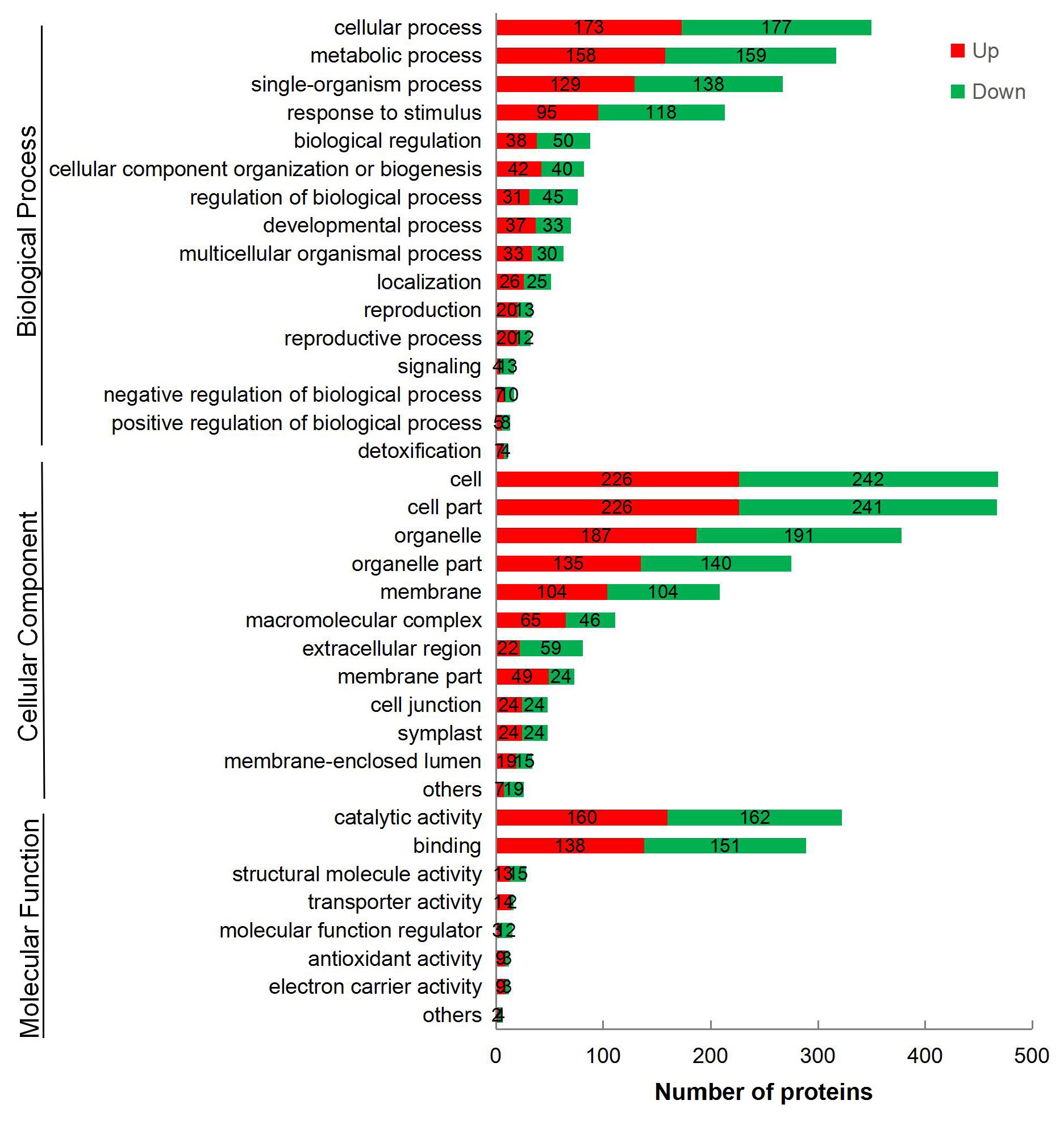
**Figure S1. GO analysis of the differentially expressed proteins in cassava leaves.** Expressed proteins involved in biological process, cellular component, and molecular function against the GO database.

**Figure S2. GO enrichment-based clustering analysis for the differentially expressed proteins.** (A)Biological process analysis; (B) Cellular component analysis; (C) Molecular function analysis.


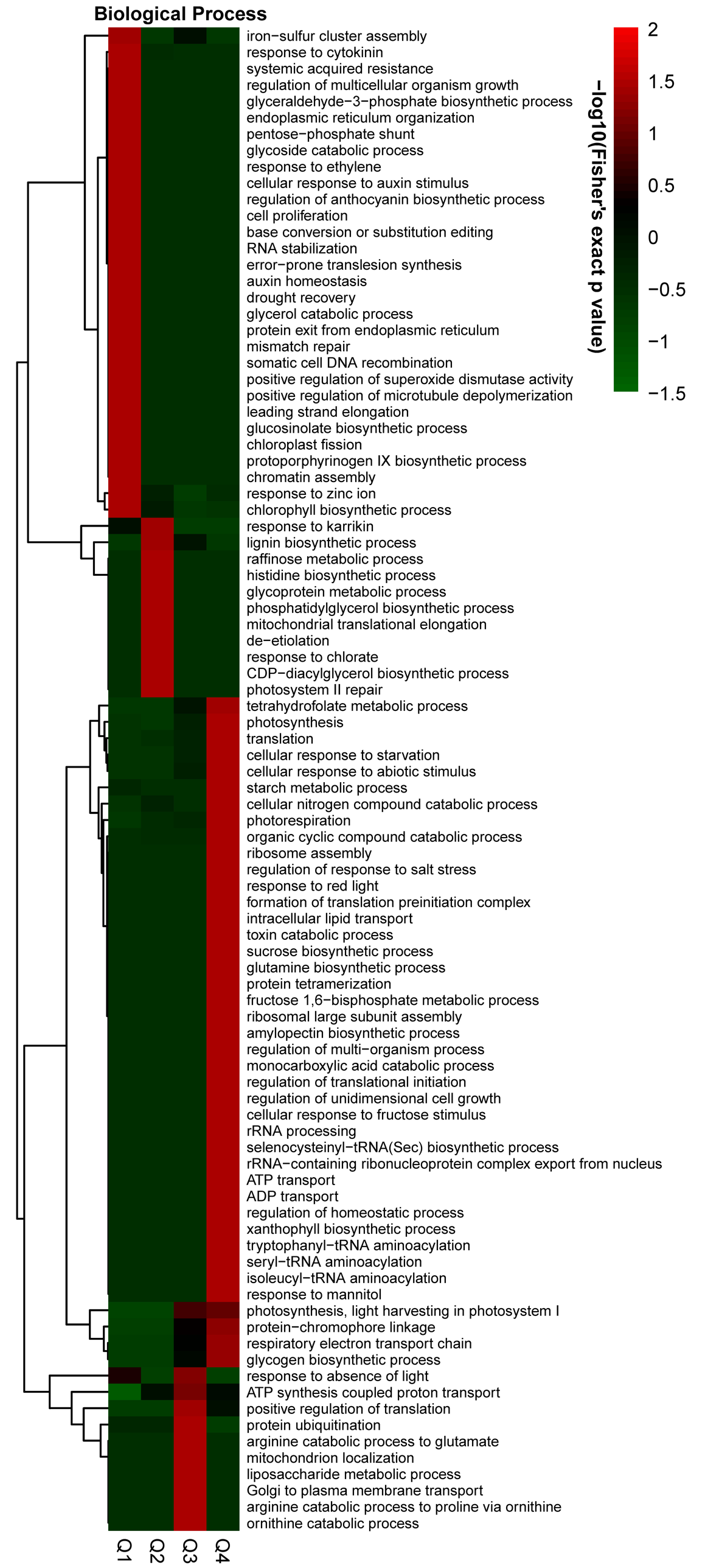


A

B


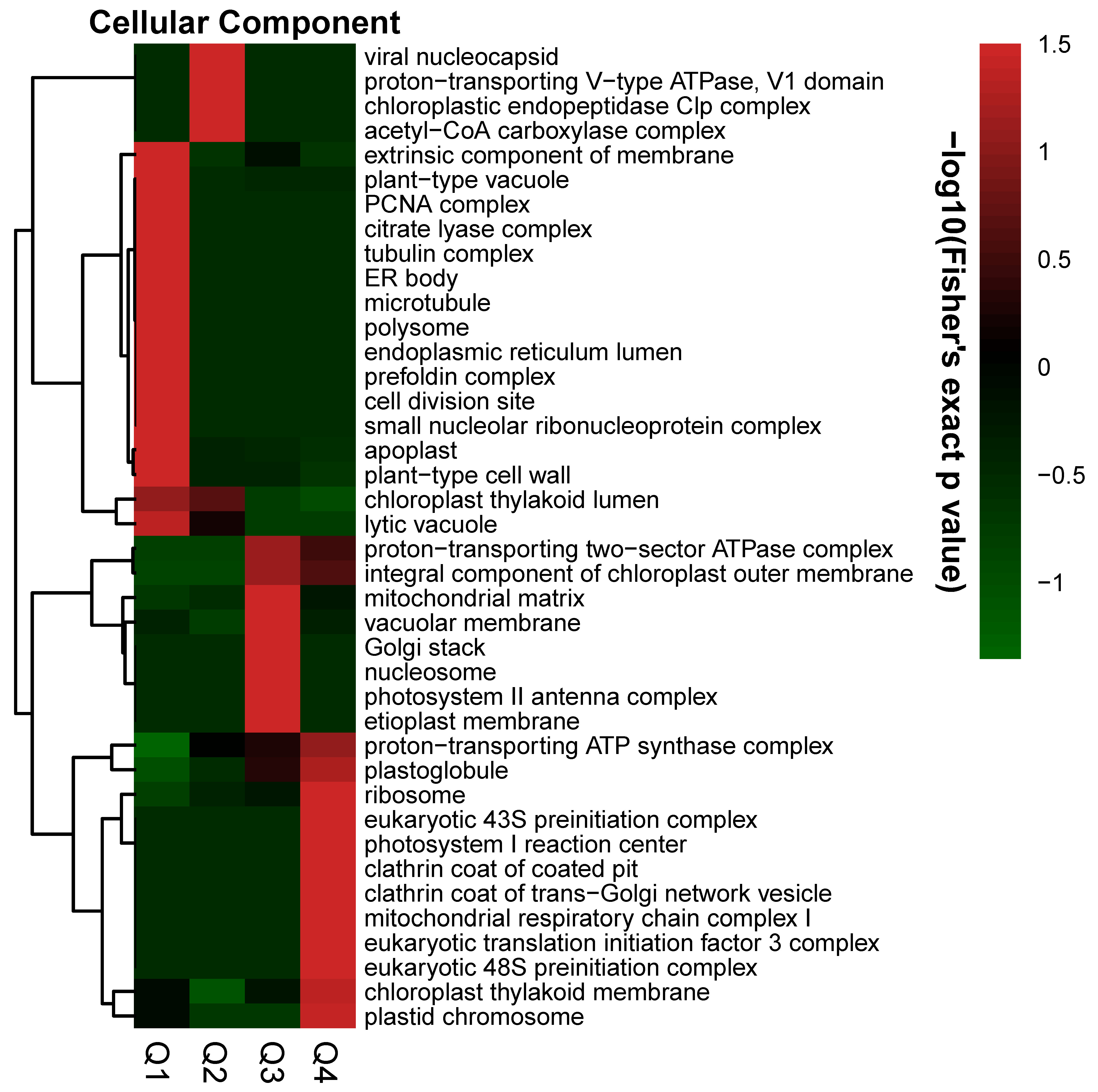

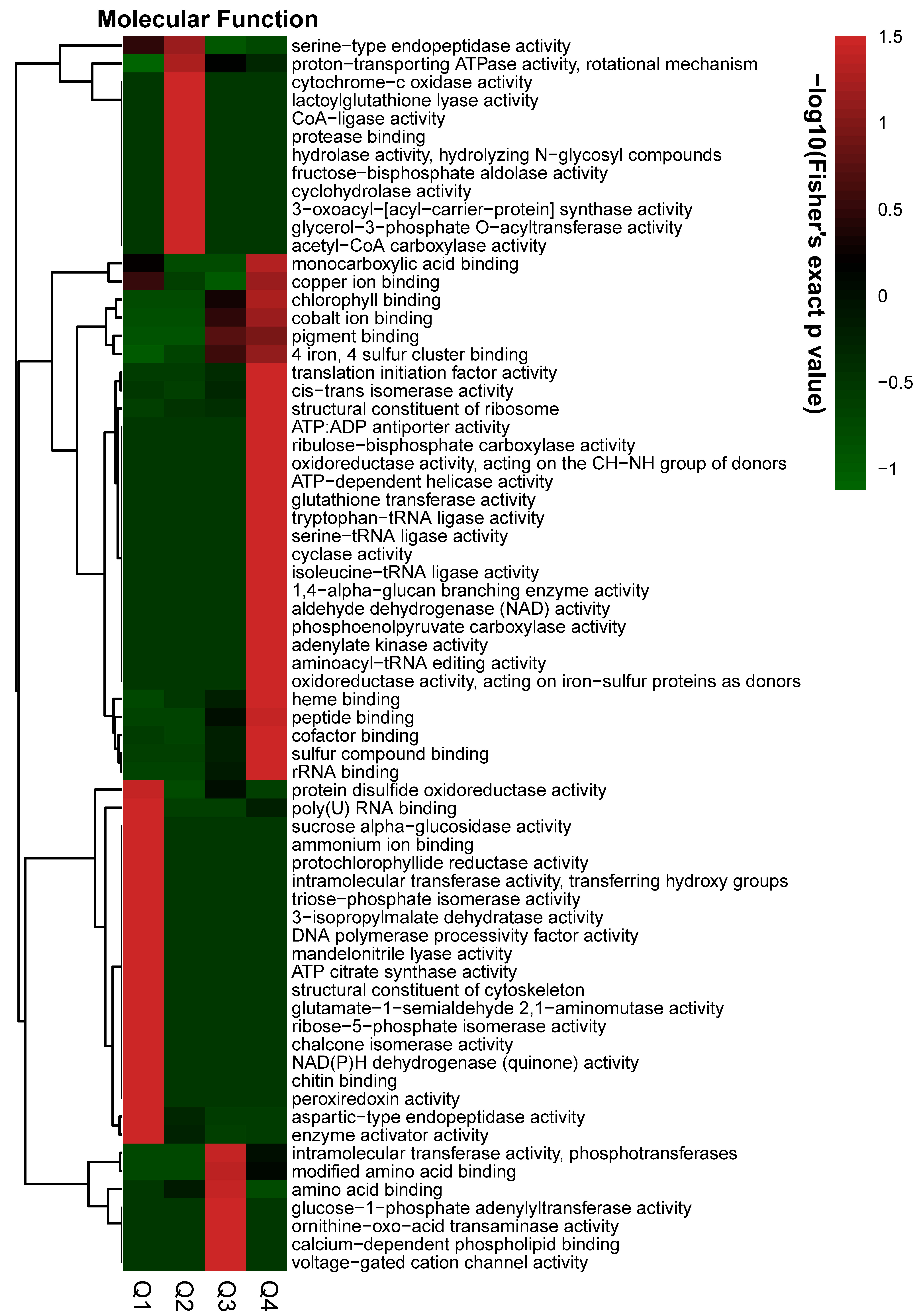


C





**Figure S3. Protein domain based clustering analysis.**
